# PI3K-Seeker: A Machine Learning-Powered Web Tool to Discover PI3K Inhibitors

**DOI:** 10.1021/acsomega.5c07315

**Published:** 2025-11-18

**Authors:** Francisca Joseli Freitas de Sousa, Dinler Amaral Antunes, Geancarlo Zanatta

**Affiliations:** § Postgraduate Programme in Biochemistry, Department of Biochemistry at Federal University of Ceará, Fortaleza 60440-554, CE, Brazil; ‡ Department of Biology and Biochemistry, University of Houston, Houston, Texas 77204, United States; † Department of Biophysics, Federal University of Rio Grande do Sul, Porto Alegre 91501-970, Brazil

## Abstract

Phosphatidylinositol 3-kinases (PI3Ks) play a crucial role in human metabolism, and their dysregulation contributes to the development of several metabolic disorders, including cancer. Despite advances in experimental high-throughput screening, discovering new therapeutic agents remains challenging and costly. In this study, we developed PI3K-Seeker, a web server based on a two-stage prediction process to find new PI3K inhibitors. The first stage eliminates nonbinders, while the second refines the selection, leaving only molecules with a high probability of being potent inhibitors. Models were trained using the XGBoost algorithm and PubChem fingerprints extracted from distinct datasets. In the first stage of classification, the model showed impressive metrics (MCC: 0.917, AUC-ROC: 0.993, and ACC: 0.917). In the second stage, the data enhancement, the model trained also performed exceptionally well (MCC: 0.939, AUC-ROC: 0.956, and ACC: 0.994). The PI3K-Seeker is a user-friendly web server suitable for a large set of compounds, available at http://www.ufrgs.br/labec/pi3k-seeker/.

## Graphical abstract

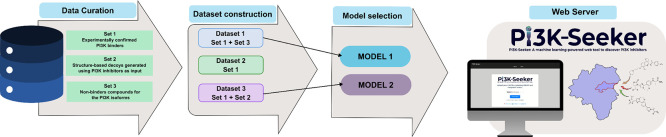

## 1 Introduction

Phosphatidylinositol 3-kinase (PI3K) is an essential family of enzymes involved in cell signaling and is responsible for an extensive range of metabolic processes. Under normal physiological conditions, the PI3K/Akt/mTOR pathway regulates cell growth and controls the cell cycle. In abnormal situations, the overactivation of PI3K is associated with various metabolic disorders and is also implicated in oncological processes.^1–4^

Class IA PI3Ks (PI3Kα, PI3Kβ, and PI3Kδ) are heterodimers composed of a catalytic subunit (p110α, p110 β, or p110 δ) and a regulatory subunit (p85). The regulatory subunit binds to phosphorylated tyrosine residues on activated receptor tyrosine kinases (RTKs). This interaction recruits the enzyme to the plasma membrane and activates the catalytic subunit, which then phosphorylates phosphatidylinositol 4,5-bisphosphate (PIP2) to generate the second messenger phosphatidylinositol (3,4,5)-trisphosphate (PIP3).^5^ Class IB PI3K (PI3Kγ) is activated downstream of G protein-coupled receptors (GPCRs). Their regulatory subunits (p101) bind directly to Gβγ subunits released from activated G-proteins, recruiting and activating the catalytic subunit at the membrane (Figure 1).^6^

**Figure 1:**
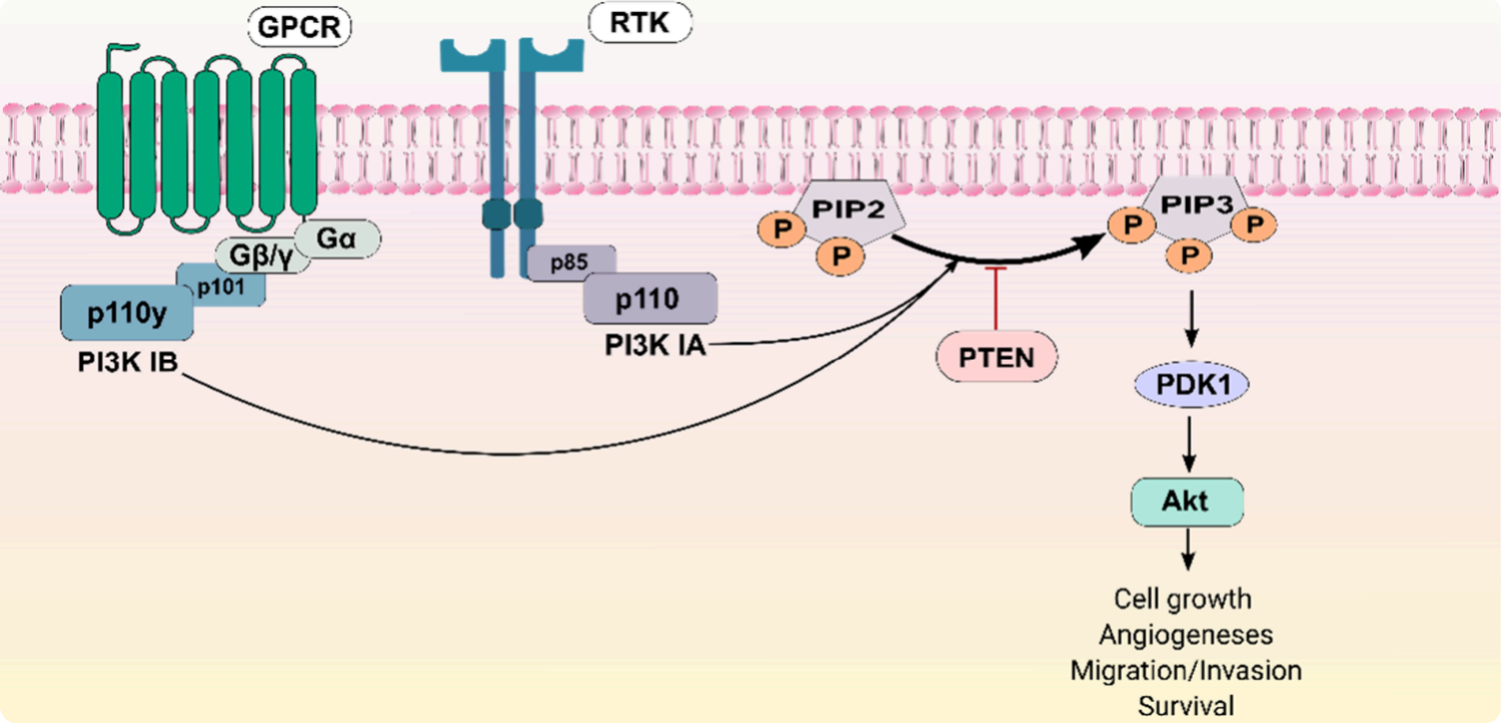
Overview of the PI3K class I pathway. The class IA PI3Ks (PI3Kα, PI3Kβ, and PI3Kδ) are activated downstream by Tyrosine Kinases (RTKs). In contrast, class IB (PI3Kγ) is activated downstream by G protein-coupled receptors (GPCRs). The catalytic subunit of PI3K phosphorylates phosphatidylinositol 4,5-bisphosphate (PIP2) to generate the second messenger phosphatidylinositol (3,4,5)-trisphosphate (PIP3). Additionally, the second messenger can be regenerated to PIP2 by the phosphatase and tensin homologue (PTEN).

PI3K class I isoforms are more studied due to their relationship with various diseases. For instance, changes in the activity of PI3Kα have been widely associated with multiple types of cancers, particularly solid tumors, as well as PIK3CA-related overgrowth spectrum (PROS) and activated phosphoinositide 3-kinase delta syndrome (APDS).^7–10^ Meanwhile, PI3Kδ and PI3Kγ isoforms are more related to immune system pathologies.^11^ Nonredundant functions associated with PI3Ks are also related to other processes directly or indirectly linked to malignancies and can have different impacts on specific types of cancers.^12,13^

Despite its importance, there is a limited number of PI3K inhibitors approved by Food and Drug Administration (FDA). Several factors contribute to this, including the incidence of serious adverse events associated with these compounds. Even among the approved inhibitors, poor tolerability, intrinsic and acquired drug resistance, and feedback signaling loops that counteract PI3K inhibition often led to treatment discontinuation.^14^ In addition, the design of new PI3K inhibitors involves several challenges, with their highly conserved ATP-binding pockets as one of the main obstacles. The PI3Kα, for example, can develop resistance to inhibition through functional compensation, or “feedback loops” via overexpression of tyrosine kinase receptors (RTKs). Other physiological changes resulting from the inhibition of this isoform may include disturbances in glucose metabolism. The use of PI3K inhibitors is associated with serious side effects resulting from their administration, such as hyperglycemia, diarrhea, nausea, pneumonia, fatigue, asthenia, skin rashes, among others, which lead to discontinuation of use or clinical trials.^15^

New technologies focused on drug discovery have been emerging, boosted by the increase in computational power and the advances in artificial intelligence (AI) approaches.^16^ In addition, the increased number of public databases of chemical and biological information including PubChem and ChEMBL facilitates the training of new models, making them a valuable source for AI applications in drug discovery.^17,18^ In this way, it is now possible to quickly scan large databases of various chemical components and use them as pharmacophores for drug design or repositioning.^19,20^

Among initiatives based on AI is the use of quantitative structure–activity relationship (QSAR) studies for the identification of ligands for specific targets. For instance, Bi et al. (2022) proposed machine learning models that utilize molecular fingerprinting to classify DYRK1A inhibitors, which are therapeutic targets for neurodegenerative diseases.^21^ Similarly, Yu et al. (2023) applied a comparable approach to CYP17A1, a target for developing anticancer molecules.^22^ The same approach was used by Yu and colleagues to identify LpxC inhibitors. In this work, they combined various fingerprints, such as MACCS and PubChem, to create classification models that predict the inhibitory activity of LpxC inhibitors.^23^ Additionally, Srisongkram and colleagues (2023) employed a combination of fingerprints with an extreme gradient boosting (XGB)-QSAR model for high-throughput screening in drug design and for the identification of KRASg12c inhibitors.^21–24^

Attempts to develop models for drug discovery targeting PI3K have also been made. In this context, Zhu and colleagues^25^ used a machine learning virtual screening approach to discover a novel selective inhibitor of PI3Kγ. Their study utilized a set of structures and ligands of the PI3Kγ isoform to construct a Naïve Bayesian Classification (NBC) model, employing a binary classification (1 for inhibitors and 0 for noninhibitors) and efficacy measured using the AUC-ROC curve (0.906). Although still an interesting approach to identify selective ligands, their approach was limited only to the isoform gamma. Recently, Kang and colleagues^26^ have expanded the toolbox by developing a deep learning model, called MVGNet, which was reported to predict inhibitory activity by classifying molecules into two categories, active and inactive, across all four PI3K isoforms (α, β, γ, and δ). While their model represents a significant advancement in addressing isoform selectivity, it still faces limitations in effectively handling the early stages of virtual screening, particularly in rapidly and accurately distinguishing PI3K binders from nonbinders within large and diverse chemical libraries. Therefore, despite these promising advances, there is still room for improvement, especially at the early stages of virtual screening, where models that combine high accuracy, broad isoform coverage, and the ability to efficiently process large-scale compound libraries are needed. Developing such models would greatly enhance the identification and prioritization of novel PI3K-targeting compounds in drug discovery pipelines.

In this work, we present PI3K-Seeker, a fast and user-friendly web server that classifies compounds as active or inactive against isoforms belonging to the PI3K class I. This tool was implemented using a two-stage pipeline based on XGB^27^ machine learning classification models. Each model was evaluated in terms of accuracy, precision, sensitivity, the Matthews correlation coefficient (MCC), and ROC AUC. As proof-of-concept, each stage of the tool was tested using real data as input, showing strong predictive performance and demonstrating its potential to accelerate the identification of novel PI3K inhibitors.

## 2 Methodology

### 2.1 Datasets

To facilitate the development and evaluation of machine learning models for the identification of PI3K class I ligands, we compiled three curated molecular sets: (set 1) experimentally confirmed PI3K binders, (set 2) structure-based decoys generated using a set of active inhibitors of PI3K as input, and (set 3) compounds as nonbinders of PI3K isoforms. Following these sets, three distinct datasets were constructed. Dataset1 comprises known binders (set 1) and nonbinders (set 3), providing a biologically grounded binary classification scenario. Dataset2 consists exclusively of the positive class (set 1) as a reference for supervised or one-class modeling approaches. Dataset3 integrates binders (set 1) and generated decoys (set 2), enabling evaluation of the capacity of the model to discriminate between actives and structurally plausible, yet presumed inactive compounds. Therefore, these datasets offer a robust framework for training and benchmarking ligand-based virtual screening algorithms across varying degrees of class separability and chemical diversity.

Set 1 was built based on data from the ChEMBL Data Web Services;^28^ source IDs were 4005, 2111367, 3130, 2111432, 3145, 3038510, 3267, and 3559703. It comprised 22,175 molecules obtained by filtering based on IC50 values and limited by species (*Homo sapiens*) and with single protein and protein complex data. Bioactivity was retrieved using IC50 values (standard unit (nM)), and missing IC50 data or duplicate values were eliminated. For clarity, IC50 values were converted into pIC50 (pIC50 = −log 10­(IC50)) and molecules were labeled as “active” when pIC50 > 6 or “inactive” when pIC50 < 5. Following data curation, 9028 unique compounds were obtained, comprising 7965 active and 1063 inactive molecules. Set 2 was built by generating decoys using the DUDE-Z server^29^ In total, 11,312 new inactive molecules were generated using this approach based on 404 active molecules from set 1. Set 3 was made of 26,019 molecules using data from ChEMBL, comprising inhibitors of apoptosis protein 3 (IAP3/BIRC3), protein kinase C beta (PKCβ), C–C chemokine receptor type 5 (CCR3), MAP kinase ERK2 (MAPK/ERK2), vascular endothelial growth factor receptor 2 (VEGFR2), Janus kinase (JAK2), hexokinase type IV, and mechanistic target of rapamycin (mTOR). mTOR inhibitors with dual activity (e.g., PI3K/mTOR) were removed. The molecules obtained from ChEMBL and decoys were processed using a PaDEL descriptor before calculating the fingerprints, with salt removal, standardization of the nitro group, and tautomer standardization.

### 2.2 Scaffold Diversity and Chemical Space

We used the RDKit to process the chemical structures of the molecules and obtain Bemis–Murcko scaffolds for active and inactive compounds. Properties such as molecular weight (MW), number of hydrogen acceptors and donors (HBA/HBD), topological polar surface area (TPSA), rotatable bond count, carbon sp3 fraction (FracCsp3), ring count, aromaticity, and violations of Lipinski’s rule of five were also calculated.^30,31^

### 2.3 Extracting Fingerprints

The molecular fingerprints used to extract features from all datasets were EState (79 bits), Molecular ACCess System (MACCS, 116 bits), and PubChem (881 bits), and PaDEL Descriptor software generated the fingerprints.^32^

### 2.4 Machine Learning Models

We compared the XGB^27^ algorithm with three others: two machine learning algorithms (Support Vector Machine, SVM, and Random Forest, RF) and one deep learning algorithm (Graph Attention Network, GAT). SVM is effective for solving classification problems by finding a hyperplane that separates different classes.^33^ Random Forest, on the other hand, utilizes an ensemble learning approach that combines multiple decision trees. In classification tasks, it determines the outcome based on a majority vote among those trees.^34^ GAT, a subtype of Graph Neural Network, allows for the identification of important nodes beyond just their structural connections within the graph. This capability helps capture complex relationships based on the content of the nodes as well.^35^

We assessed metrics such as accuracy, precision, recall, F1-score, Matthews Correlation Coefficient (MCC), and ROC AUC (area under the curve of the receiver operating characteristic). We divided each dataset into a training set (80%) and a test set (20%). We performed a comprehensive 10-fold cross-validation to mitigate the possibility of overfitting.^36^ The hyperparameter values for each method trained are described in Supplementary Table S1, while the evaluation metrics are provided in Supplementary Table S2.

### 2.5 Assessment of Machine Learning and Fingerprint Outcomes

Furthermore, the models were evaluated to assess their performance across various metrics, including accuracy, precision, recall, F1-score, ROC AUC, and the Matthews Correlation Coefficient (MCC). These metrics were computed for test sets to comprehensively gauge the efficacy of each model.^37,38^ In addition, the SHapley Additive exPlanations (SHAP) tool helped to understand how the model interprets data.^39^

To define an applicability threshold, we define an applicability domain using the LOF (local outlier factor) method. LOF stands out for comparing a point with its immediate neighborhood, being more sensitive to outliers that global methods would not notice.^40^

### 2.6 Machine Learning-Powered Web Tool to Discover PI3K Inhibitors—PI3K-Seeker

The PI3K-Seeker server was built to analyze the input data in two stages. In the first stage, it employs ML model 1, which was trained with dataset1 and eliminates non-PI3K binders. In the second stage of the analysis, ML model 2, which was trained with dataset3, classifies the remaining compounds as weak or strong binders, making the final prediction. PI3K-Seeker initially works with a CSV file provided by the users that includes a column naming the molecules and the molecules themselves in the SMILES format. After submitting the input files, PI3K-Seeker does the predictions for each molecule and returns it as “active” or “inactive”. All data used in the training of the models are available in the Supporting Information. The process of creating datasets leading up to the development of models is illustrated in Figure 2.

**Figure 2:**
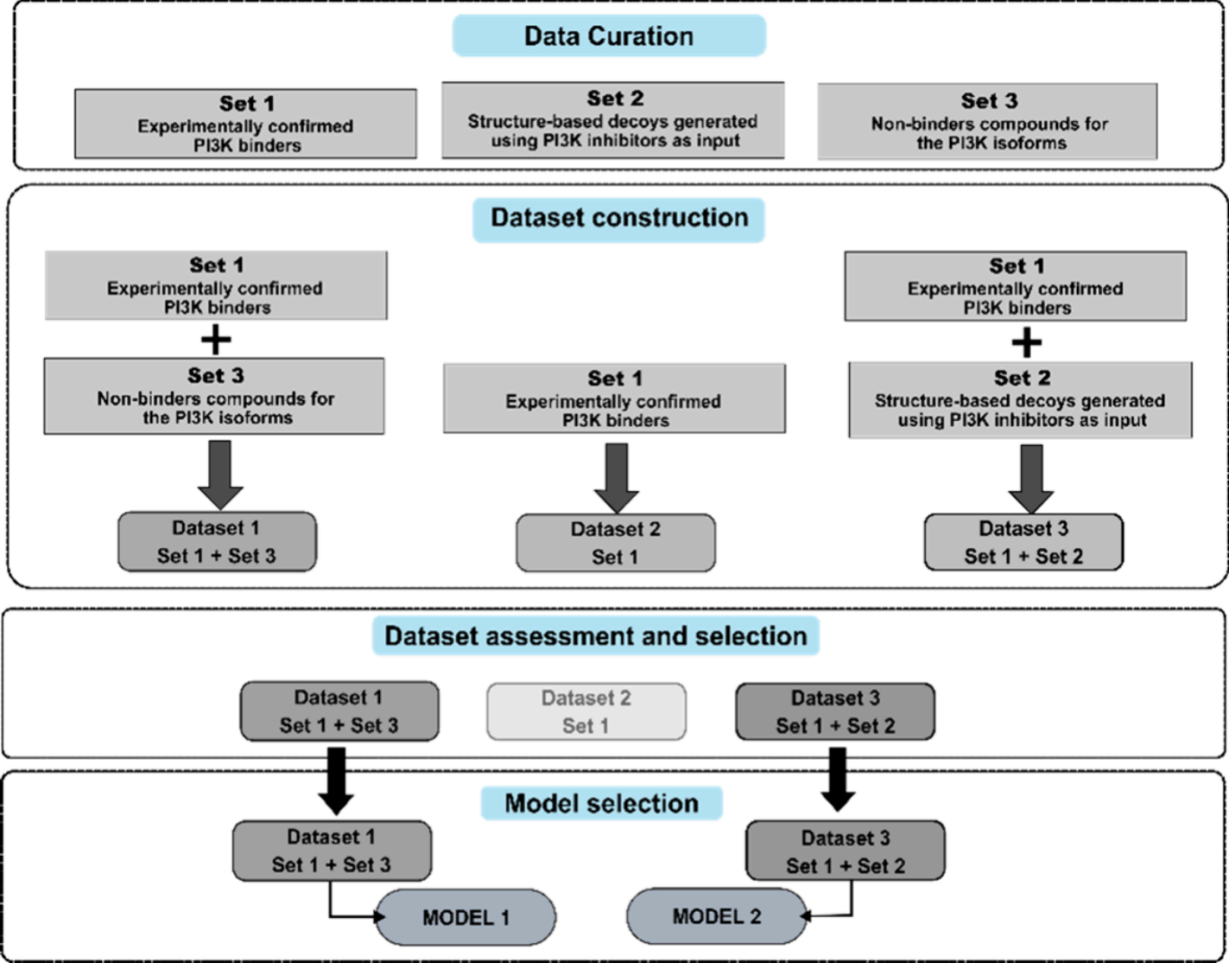
Flowchart showing the path from data curation to model selection.

## 3 Results and Discussion

### 3.1 Scaffold Diversity and Chemical Space

We used the RDKit package to process the chemical structures of the molecules and obtain Bemis–Murcko scaffolds for active and inactive compounds. Properties such as molecular weight (MW), number of hydrogen acceptors and donors (HBA/HBD), topological polar surface area (TPSA), rotatable bond count, sp3 carbon fraction (FracCsp3), ring count, aromaticity, and violations of Lipinski’s rule of five were also calculated.

Scaffold analysis was conducted using the Bemis–Murcko framework on all molecules included in model training, categorized as active or inactive. Figure 3A details the frequency of the five most common scaffolds within the inactive compounds, with their respective structures on the right. In contrast, Figure 3B shows the most common scaffolds in the active compounds. Although fewer in number, the active scaffolds are structurally more complex than those in inactive compounds, including motifs such as morpholine, sulfonamide, and dihydroquinazolin-4-one. The uniform manifold approximation and projection (UMAP) analysis in Figure 3C illustrates clusters of molecules with similar physicochemical profiles. Despite the presence of outliers, active and inactive compounds exhibit considerable overlap, with no clear separation between the classes, highlighting the importance of applying machine learning models capable of capturing more subtle patterns. Details of physicochemical properties are shown in Supplementary Figure S1.

**Figure 3:**
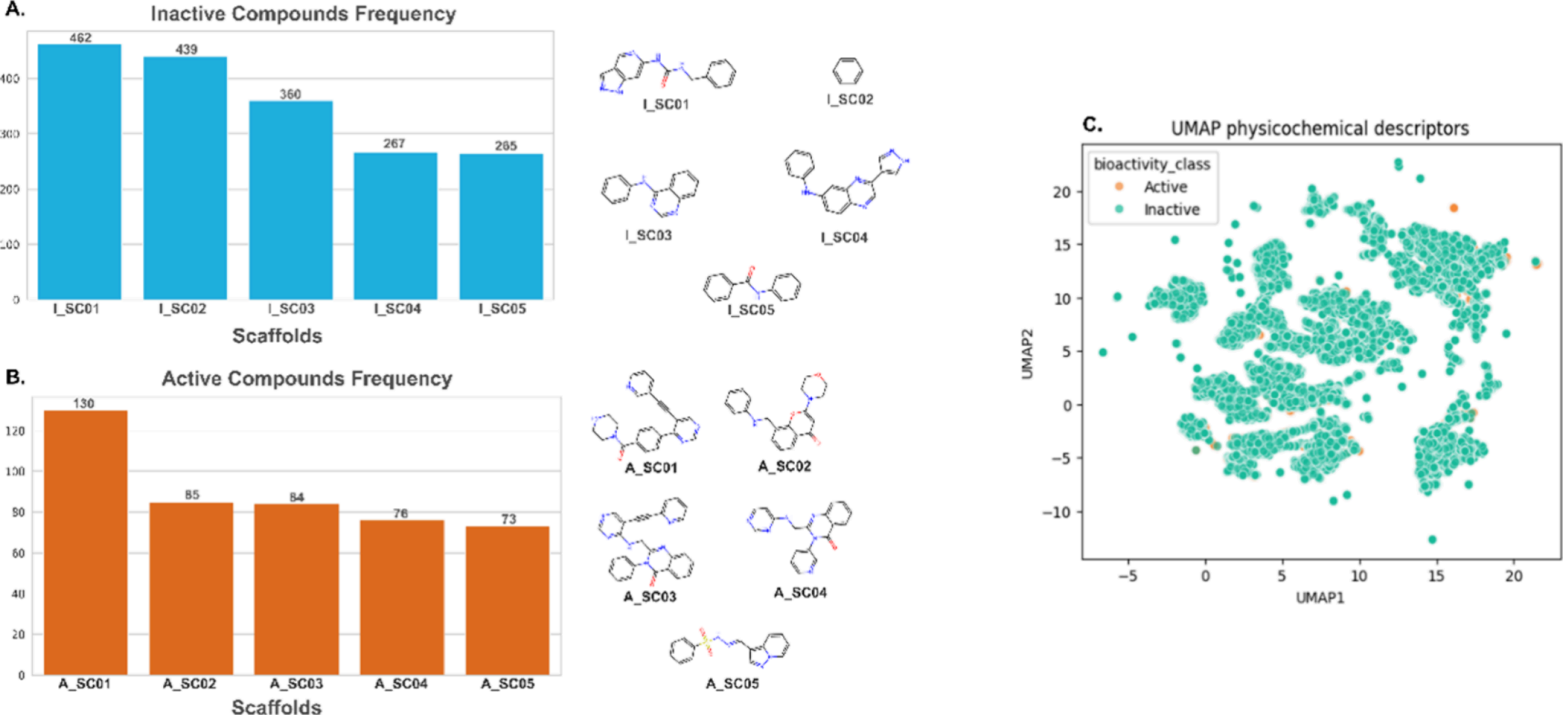
Scaffold diversity and chemical space. (A) Inactive compound frequency and (B) active compound frequency. (C) UMAP showing physicochemical descriptors in a 2D representation.

### 3.2 Machine Learning Models

We tested a different combination of fingerprints and algorithms. As Figure 4 shows, the best-performing fingerprint was PubChem for each dataset. The best-performing algorithms were XGB and SVM. The complete metrics are presented in Table S2 of the Supporting Information.

**Figure 4:**
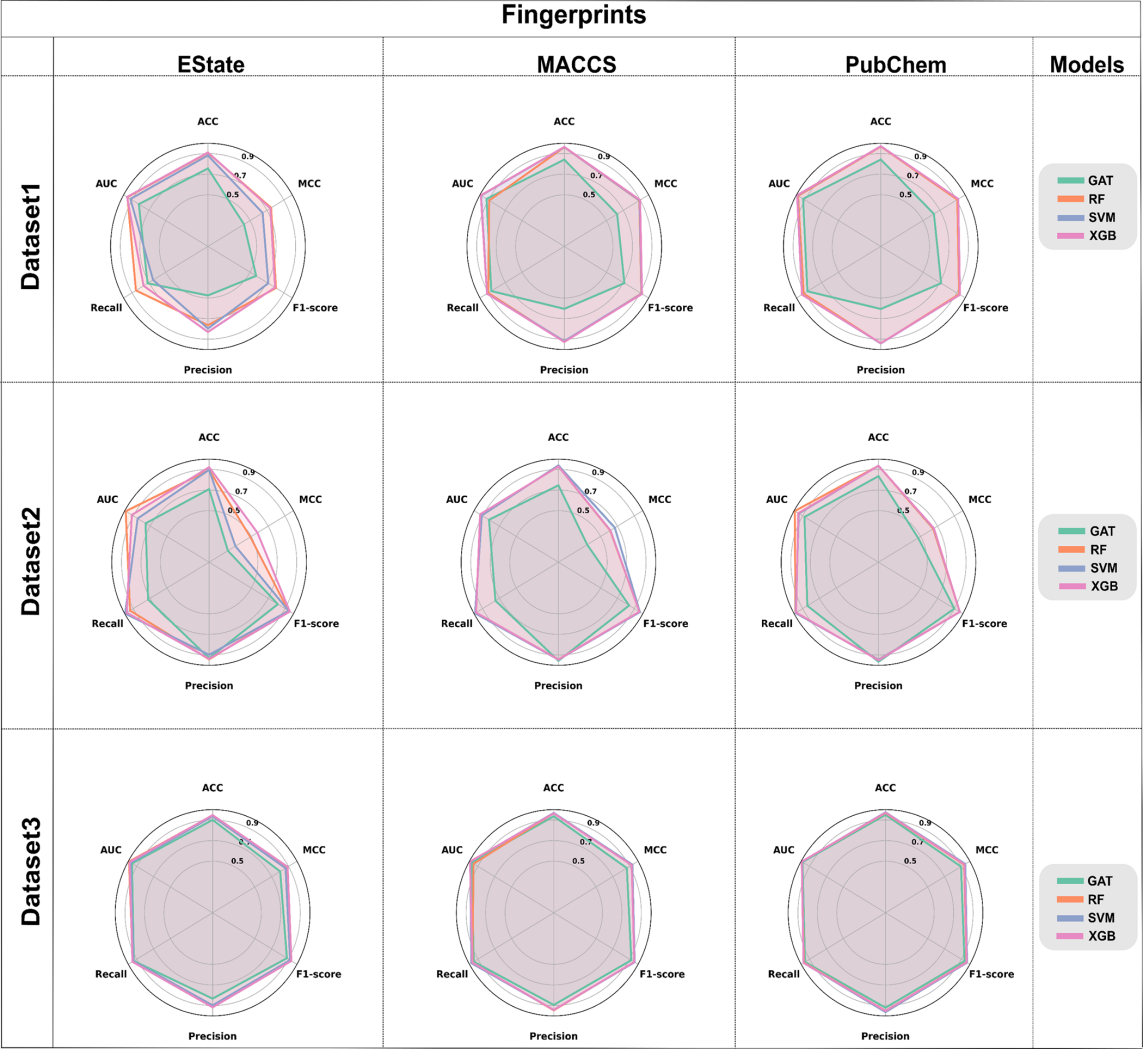
Metrics results of models tested. Legend: SVM (support vector machine), RF (random forest), XGB (extreme gradient boosting), GAT (graph attention networks), and three fingerprints (EState, MACCS, and PubChem).

According to Figure 4, when models were trained with dataset1, XGB, RF, and SVM methods showed superior performance using PubChem as fingerprint. The use of dataset1 highlights the balance of metrics, all greater than 0.9. GAT did not perform satisfactorily when compared to the others, especially in terms of accuracy, F1-score, and MCC. MCC is a more comprehensive metric because it considers the four quadrants of the confusion matrix (TP, FN, TN, FP), measuring the correlation between actual labels and predicted labels.^38^ The MACCS fingerprint had good metrics with the SVM and XGB algorithms, not surpassing PubChem, but better than EState. Overall, the algorithms performed worst on dataset2, especially when analyzing MCC values. For EState, from best to worst, the order was XGB > RF > SVM > GAT; for MACCS, SVM had a higher MCC value, and the order from best to worst was SVM > XGB > RF > GAT. Interestingly, all algorithms performed excellently with dataset3. Despite using approximate values, the PubChem fingerprint showed the best results overall. Among the algorithms, XGB and SVM performed exceptionally well. The results from the XGB were consistent across various datasets and fingerprints, consistently ranking among the best, particularly for MCC values. Due to this reliability, we opted to include XGB in the server pipeline.

To enhance the efficiency and predictive performance of the platform, we implemented a two-stage virtual screening strategy. In the first stage, a fast and accurate classification model was employed to eliminate compounds with a low likelihood of binding to PI3K, thereby significantly reducing the chemical search space. This initial filter was built using an XGB model trained on dataset1, which included both known PI3K binders and nonbinders. As shown in Table 1, the model exhibited strong predictive performance and generalizability, making it well-suited for the initial filtering step.

**Table 1:** Metrics for Dataset1 and PubChem Fingerprints Are Implemented in the First Step of the Virtual Screening Pipeline

parameter	metric	value
training	MCC_train	0.987
	CV_mean	0.971
	CV_SD	0.003
test	MCC_test	0.919
	precision	0.939
	accuracy	0.971
	recall	0.936
	F1-score	0.937
	AUC-ROC	0.993

In the second stage, more computationally intensive analyses were performed on the subset of compounds retained from the initial filtering, allowing for a more refined identification of high-confidence PI3K binders. This stage required a model able to distinguish not only between binders and nonbinders but also to capture differences in the predicted binding affinity. To achieve this, we tested the performance of two XGB models. The first model was trained on dataset2, which includes only known PI3K binders, categorized as “active” or “inactive” based on their pIC50 values. The second was trained on dataset3, which expanded the negative class by incorporating a large number of decoy compounds alongside the active ligands from dataset2, thereby enhancing the capacity of the model to minimize the prediction of false positives.^41^

As the model trained on dataset3 outperformed the one trained on dataset2, it was implemented in the second stage of the server’s analysis. The improvement obtained with this model is reflected across multiple performance metrics, as shown in Table 2. Although MCC was considered for model selection, other metrics, such as precision, recall, and AUC-ROC, were also critical for evaluating the ability of the model to generalize and correctly classify both active and inactive compounds. A comparative overview of the results is presented in Table 2.

**Table 2:** Metrics for XGB and PubChem Fingerprints Models Trained for the Second Step of the Virtual Screening Pipeline

dataset split	metrics	XGB (dataset2)	XGB (dataset3)
training	CV_mean	0.939	0.972
	CV_SD	0.006	0.005
	MCC_train	0.985	0.996
test	MCC_test	0.658	**0.937**
	precision	0.951	**0.952**
	accuracy	0.935	**0.971**
	recall	**0.977**	0.972
	F1-score	**0.964**	0.962
	AUC-ROC	0.943	**0.993**

### 3.3 Interpreting the Predictions Made by the Models

Subsequently, to enhance interpretability and elucidate the decision-making process of the model, we employed SHAP (SHapley Additive exPlanations), a widely recognized framework for interpreting complex machine learning models.^39,42^ SHAP analysis enabled the identification of key molecular substructures that drive prediction outcomes, thereby increasing confidence in both the interpretability and practical applicability of the model.

As shown in Figure 5, the XGB model trained on dataset1 (model 1), the descriptor with the most significant impact was PubchemFP392, which corresponds to a secondary amine (R-NH-R′). The SHAP analysis of the impact of this feature showed that its high frequency is strongly associated with positive SHAP values. This indicates that the model has learned to recognize this group as a strong predictor of inactivity. The model showed a tendency to penalize simple fragments, which is reinforced by the features PubchemFP635 (hydrazine, for example, Figure 5) and PubchemFP791 (1,4-diaminacyclohexane, for example, Figure 5), which shift the prediction to the inactive class. Taken together, these results suggest that the model has learned to filter out molecules containing nitrogenous and low-density groups that are atypical in PI3K inhibitors. Other fingerprint features, such as PubChemFP193, FP728, FP368, and FP335, also contributed to the prediction of nonbinders, further reinforcing the discriminatory capacity of the model for early stage filtering of inactive molecules.

**Figure 5:**
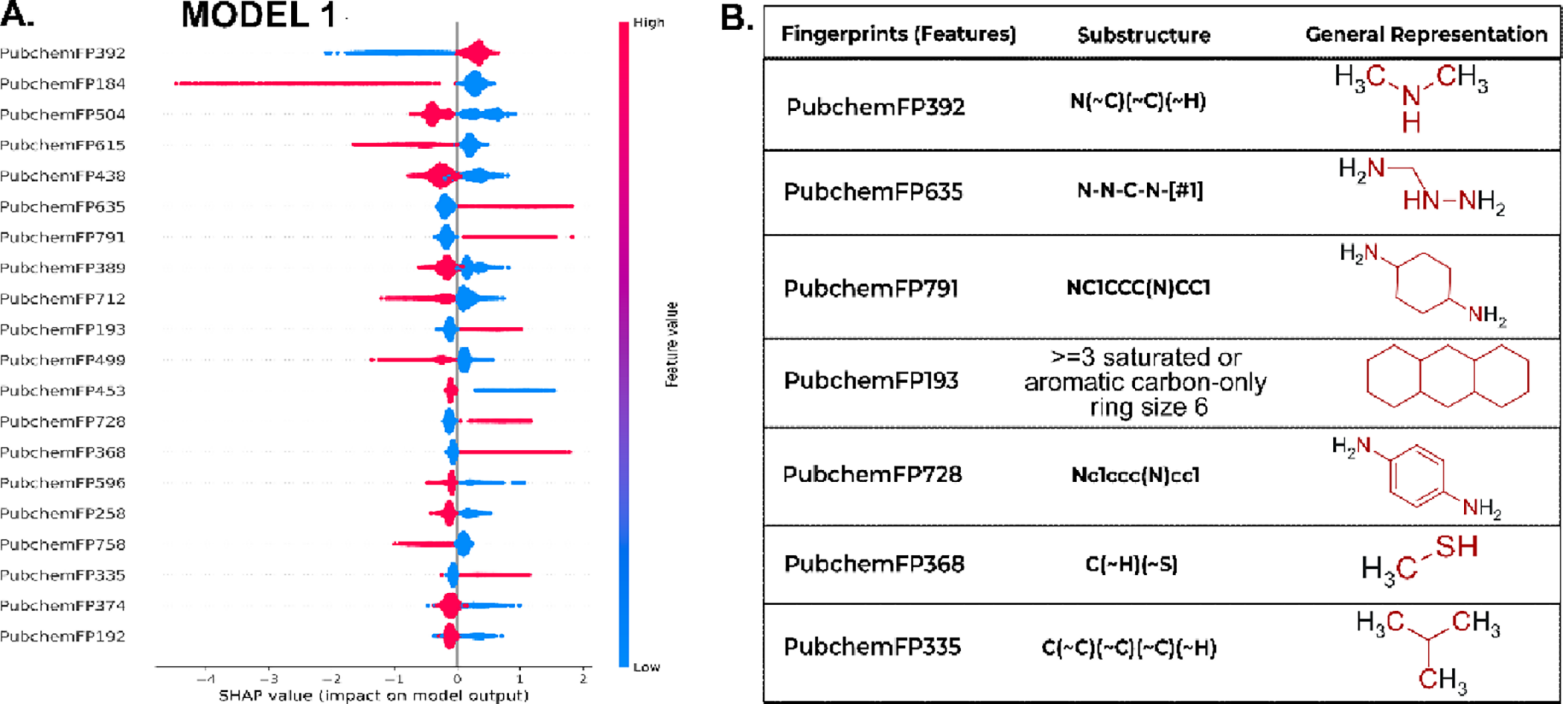
SHAP plot of model 1. In panel (A), the features were ranked according to the extent of their influence on the decisions of the models. Panel (B) shows the patterns associated with the prediction of PI3K noninhibitors.

In contrast, the XGB model trained on dataset3 (model 2), which aims to differentiate between weak and strong PI3K binders in the second stage of analysis performed by the server, exhibited a set of features with high values in the left (associated with active compounds). To illustrate how model 2 identifies active compounds, we used the known PI3K inhibitor, Buparlisib, as a case study for interpreting SHAP results (Figure 6). The functional group feature with the greatest impact, PubchemFP181, represents the presence of six-membered rings, saturated or aromatic, containing heteroatoms. Buparlisib exemplifies this characteristic very well, as its structure features pyridine and pyrimidine rings, along with two morpholine units (Figure 6B). SHAP analysis confirms that the presence of these fragments results in a strong negative SHAP value, validating them as key indicators in the prediction of active compounds. This structural signature is complemented by other important functional group feature, such as PubchemFP192 (three or more rings of size 6), reflecting the polycyclic nature of inhibitors in the classification of active compounds. Similarly, the high nitrogen count, captured by PubchemFP16 (four or more N atoms), also drives the prediction to “active”, which is consistent with the profile of Buparlisib. Taken together, the analysis of Buparlisib reveals that the model has learned to associate activity with a more complex chemical structure, characterized by nitrogen-rich systems with multiple heteroatoms in six-membered rings.

**Figure 6:**
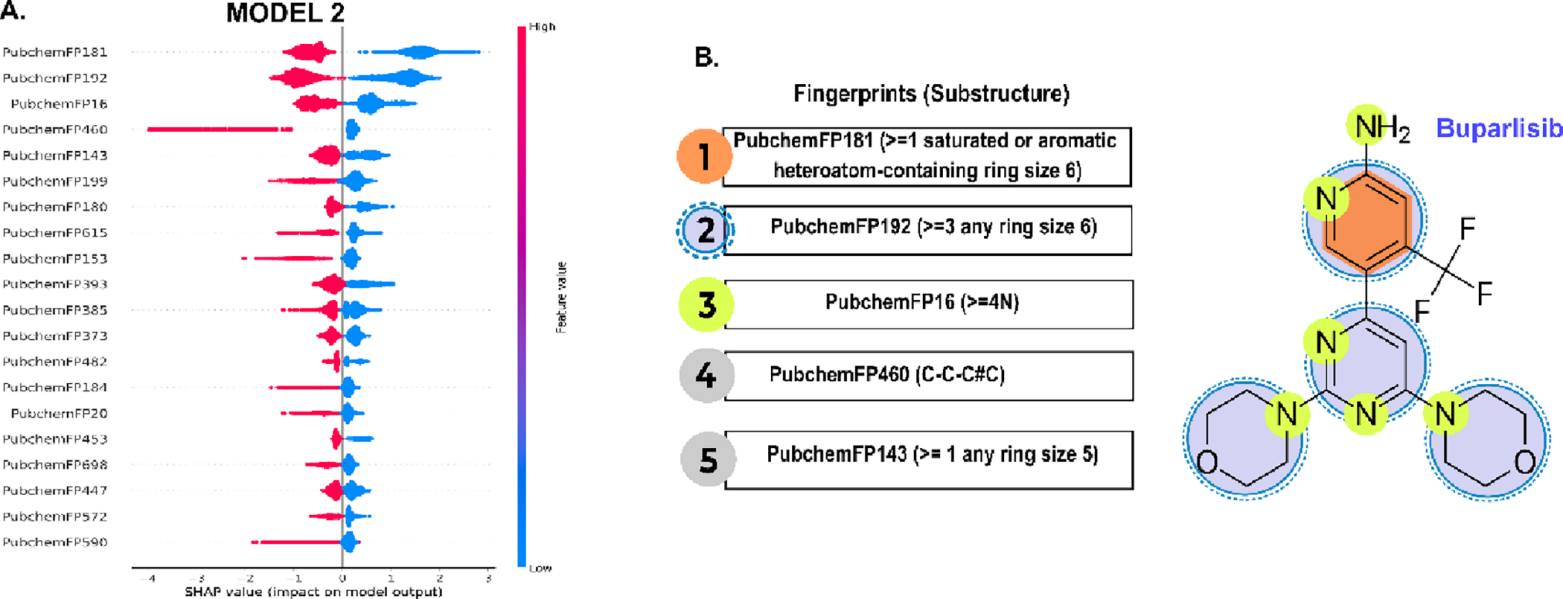
Fingerprints and substructure patterns. (A) SHAP plot associated with the active classification of model 2. (B) Fingerprints and the substructures that they represent are numbered 1–5, along with a description. In compound Buparlisib, a pan-inhibitor of PI3K, the substructures are highlighted. Each number corresponds to a color shown in Buparlisib, and they are linked to the classification of active compounds.

Interestingly, the two models shared a few features, and their contributions varied significantly in both direction and magnitude. This divergence underscores the complementary roles of models 1 and 2 within the classification pipeline, enhancing both the precision and efficiency of PI3K inhibitor identification across chemically diverse compound libraries.

### 3.4 Applicability Domain (AD)

Our models underwent a two-stage validation process by applying AD in both model 1 and model 2. First, we demonstrated its robustness and generalization ability in a chemically diverse dataset (dataset1, model 1), as shown in Table 3, maintaining high performance even for samples outside the domain of applicability. Next, we proved its remarkable sensitivity and discriminatory efficiency in a rigorous test with decoys (dataset3, model 2), confirming its effectiveness for the critical task of refining PI3K ligands. This dual approach with AD ensures that the model is not only a reliable generalist but also a high-performance specialist, validating it for practical application.

**Table 3:** Applying the Applicability Domain (AD) in Model 1 and Model 2

	XGB-dataset1 (test)		XGB-dataset3 (test)	
metrics	in AD	out AD	in AD	out AD
MCC	**0.920**	0.906	**0.941**	0.856
accuracy	0.972	0.969	0.972	0.931
precision	0.939	0.944	0.955	0.917
recall	0.937	0.907	0.972	0.976
F1-score	0.938	0.925	0.964	0.945

### 3.5 Web Server Deployment

The PI3K-Seeker web server is a user-friendly and computationally efficient platform designed to accelerate the identification of new inhibitors targeting class I PI3Ks. Implemented in Python, the application employs the XGB algorithm to predict the ability of small-molecule ligands to bind to the PI3K isoforms α, β, γ, and δ. The only required input is a list of SMILES strings representing the chemical structures of the candidate molecules to be evaluated (Figure 7A).

**Figure 7:**
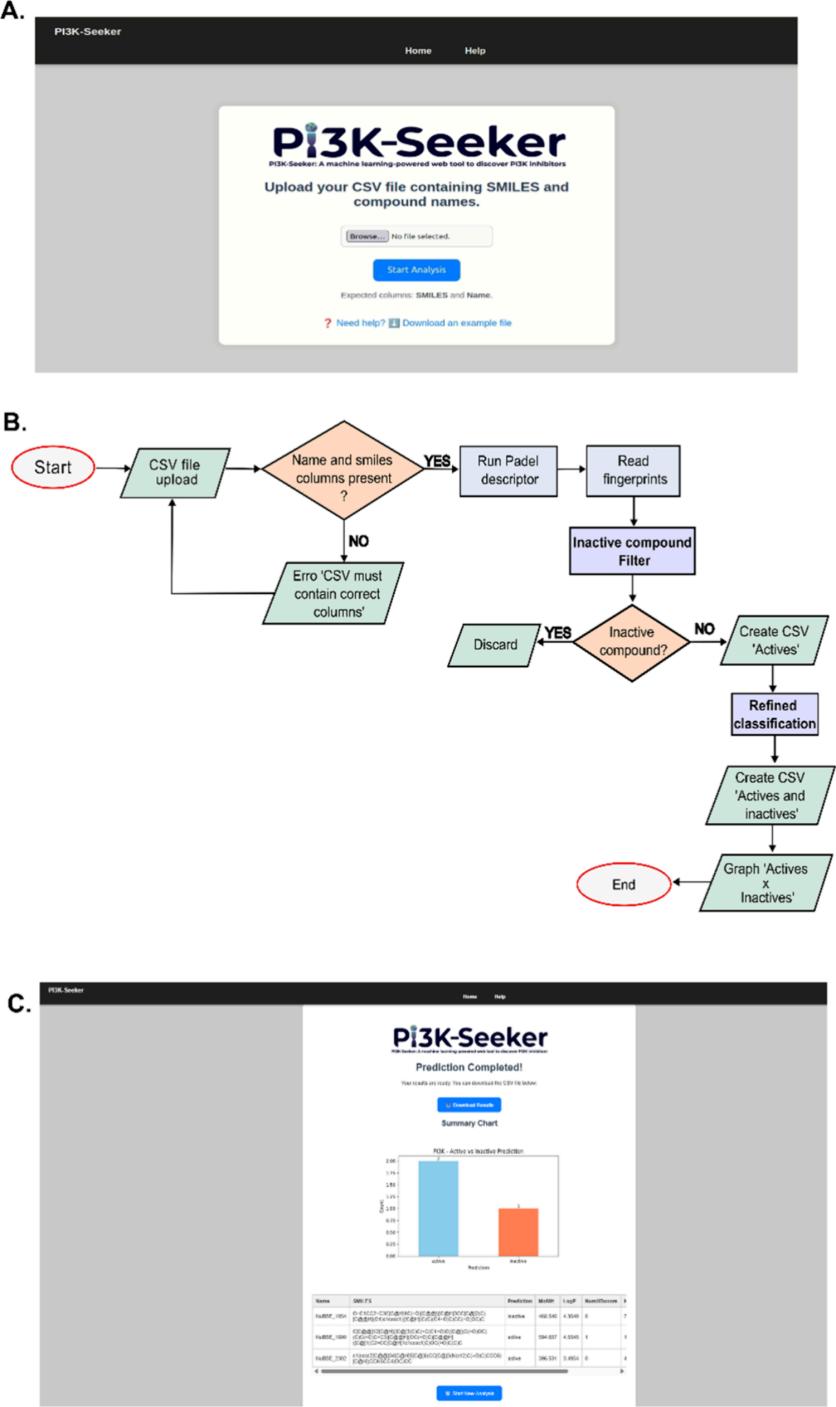
General overview of the PI3K-Seeker web server. (A) Input screen; (B) workflow including data processing, classification, and results generation; and (C) the output page showing a graphical representation of results and links to download CSV files with result data.

Initially, the algorithm performs a validation step to ensure that the input file contains exclusively SMILES representations of small-molecule ligands. Subsequently, the platform computes molecular fingerprints using the PubChem fingerprinting methodology, executes predictive modeling, and generates the corresponding output files (Figure 7B). The results page provides access to a CSV file containing detailed information for each compound (Figure 7C), including the molecule name, SMILES string, predicted activity, molecular weight (MW), LogP, number of hydrogen bond donors (HBD), number of hydrogen bond acceptors (HBA), and Topological Polar Surface Area (TPSA). The entire workflow is computationally efficient, requiring only a fraction of a second per molecule.

The high processing speed of PI3K-Seeker comes mainly from the use of the XGB algorithm as the core predictive model. Designed to efficiently handle high-dimensional and sparse datasets, such as those encountered in biological and cheminformatics applications, XGB supports parallelization across CPUs and GPUs, enabling rapid execution at scale.^27^ Its scalability and predictive accuracy have been extensively validated across various biomedical contexts, including quantitative structure–activity relationship (QSAR) modeling, classification of early and late-stage cancers, and the prediction of clinical treatment outcomes, acute kidney injury prediction, blood–brain barrier drug classification, and biomarker discovery in Alzheimer’s disease transcriptomic data.^43–46^

### 3.6 Proof-of-Concept Validation

To better evaluate the performance of PI3K-Seeker, we subjected it to two distinct and challenging real-world scenarios. In the first case study, we assessed the server using PI3K ligands extracted from crystallographic structures, also absent from the training set.

Also, we compared it with SVM, which performed very similarly to XGB using the parameters applied in PI3K-Seeker. In the second case study, the PI3K-Seeker was tested with a dataset composed exclusively of non-PI3K binders retrieved from the ChEMBL database, which were not included in the training data of the model.

As shown in Table 4, the PI3K-Seeker server was evaluated for its ability to classify ligands from crystallographic PI3K structures not used during model training. A total of 14 ligands were tested: 11 with confirmed inhibitory activity against at least one PI3K isoform and three classified as noninhibitors. As the primary objective of this test was to assess the capacity of the model to distinguish between high- and low-affinity binders, crystallographic noninhibitors were included as controls based on their well-characterized interactions with key residues in the PI3K binding site. Among them, ligand L2 V interacts with residues such as R770α and W780α, critical for PI3Kα isoform selectivity, without interfering with kinase activity, thereby validating its classification as a noninhibitor.^47^ Similarly, PBU, a di-C4-phosphatidylinositol-4,5-bisphosphate (diC4-PIP2) lipid substrate mimetic used to study PI3Kα catalysis, was correctly identified as a noninhibitor.^48^ The ATR inhibitor 8DV, structurally distinct and inactive against PI3K, was also correctly classified.^49^

**Table 4:** Ligands Found in the Crystallographic Structures of PI3K and Tested with PI3K-Seeker versus SVM

PDB	ligand	IUPAC	state	prediction PI3K-Seeker	prediction SVM
3PRZ^50^	3RZ	4-amino-2-methyl-*N*-(1*H*-pyrazol-3-yl)quinazoline-8-carboxamide	active	**active**	**active**
3PS6 ^50^	3PS	4-amino-*N*-(6-methoxypyridin-3-yl)-2-methylquinazoline-8-carboxamide	active	**active**	**inactive**
4OVV^48^	PBU	[(2*R*)-2-butanoyloxy-3-[hydroxy-[(1*R*,2*R*,3*S*,4*R*,5*R*,6*S*)-2,3,6-trihydroxy-4,5-diphosphonooxycyclohexyl]oxyphosphoryl]oxypropyl] butanoate	inactive	**inactive**	**inactive**
5JHA^51^	6K7	[1-{4-[6-amino-4-(trifluoromethyl)pyridin-3-yl]-6-(morpholin-4-yl)pyrimidin-2-yl}-3-(chloromethyl)azetidin-3-yl]methanol	active	**active**	**inactive**
5UK8 ^49^	8DV	(*R*)-4-(6-(1-(cyclopropylsulfonyl)cyclopropyl)-2-(1*H*-indol-4-yl)pyrimidin-4-yl)-3-methylmorpholine	inactive	**inactive**	**inactive**
5XGH^52^	84U	3-[(4-fluorophenyl)methylamino]-5-(4-morpholin-4-ylthieno[3,2-*d*]pyrimidin-2-yl)phenol	active	**active**	**active**
6EYZ^53^	C5W	2-methoxy-5-[4-[5-[(4-propan-2-ylpiperazin-1-yl)methyl]-1,3-oxazol-2-yl]-2∼{*H*}-indazol-6-yl]pyridine-3-carboxylic acid	active	**active**	**active**
6GY0 ^54^	FGE	∼{*N*}-[4-methyl-5-(1-oxidanylidene-7-sulfamoyl-isoindol-5-yl)-1,3-thiazol-2-yl]ethanamide	active	**active**	**active**
6ZAA^55^	QD2	4-[6-methoxy-5-(methylsulfamoyl)pyridin-3-yl]-∼{*N*}-(1-methylpiperidin-4-yl)-2,3-dihydro-1,4-benzoxazine-6-carboxamide	active	**active**	**active**
8AM0 ^56^	MWF	(2*R*)-2-[[2-[(4*S*)-4-[bis(fluoranyl)methyl]-2-oxidanylidene-1,3-oxazolidin-3-yl]-5,6-dihydroimidazo[1,2-*d*][1,4]benzoxazepin-9-yl]amino]propanamide	active	**inactive**	**inactive**
8ILV^47^	L2V	*N*-[(2*R*)-1-(ethylamino)-1-oxidanylidene-3-[3-(2-quinoxalin-6-ylethynyl)phenyl]propan-2-yl]-2,3-dimethyl-quinoxaline-6-carboxamide	inactive	**inactive**	**inactive**
8SC8 ^57^	D0D	*N*-[(5*P*)-2-chloro-5-(4-{[(1*R*)-1-phenylethyl]amino}quinazolin-6-yl)pyridin-3-yl]methanesulfonamide	active	**active**	**active**
9GCF^58^	A1IJ5	3-[(1*S*)-1-[4-azanyl-3-(5-oxidanylpyridin-3-yl)pyrazolo[3,4-*d*]pyrimidin-1-yl]ethyl]-4-[3-[(4-methylpiperazin-1-yl)methyl]phenyl]isochromen-1-one	active	**active**	**active**
9GDI^58^	A1IJ1	3-[(1*S*)-1-[4-azanyl-3-(3-fluoranyl-5-oxidanyl-phenyl)pyrazolo[3,4-*d*]pyrimidin-1-yl]ethyl]-4-(1-methyl-3,6-dihydro-2*H*-pyridin-4-yl)isochromen-1-one	active	**active**	**active**

As shown in Table 4, PI3K-Seeker achieved correct classification for 13 of the 14 compounds. The only misclassified ligand was MWF (Inavolisib), a molecule that does not act through conventional kinase inhibition but instead induces proteasome-dependent degradation of the mutant p110α protein.^56^ This mechanistic divergence from the training data likely accounts for the misclassification. Inavolisib was approved by the U.S. Food and Drug Administration in October 2024, in combination with palbociclib and fulvestrant, for treating endocrine-resistant, PIK3CA-mutated, hormone receptor-positive, HER2-negative advanced breast cancer.^27^

When dealing with external data with PI3K inhibitors, SVM ended up incorrectly classifying active inhibitors as inactive. Therefore, the underperformance below expectations is a bottleneck that prevents the expected improvement in phase two. In this regard, XGB proved to be superior, capable of satisfactorily separating inactive ligands and identifying active ones. However, both algorithms failed to classify Inavolisib. The final choice of pipeline was based on the balance between robustness and accuracy. In this scenario, we have XGB (PI3K-Seeker) as the alternative that best balances these aspects.

In the second case study, the server demonstrated excellent performance in correctly identifying non-PI3K binders across a diverse set of protein targets, with predictive accuracy exceeding 95% in the vast majority of datasets (Table 5). This high accuracy confirms the model’s ability to effectively discriminate PI3K-specific ligands from those active against unrelated molecular targets, even within large and structurally diverse compound libraries. Notably, the server maintained high discriminatory efficiency for other kinase targets, such as EGFR (97.9%), FGFR1 (94.8%), RAF (96.0%), and insulin receptor (98.5%), reinforcing its robustness in distinguishing PI3K inhibitors from other ATP-competitive ligands. Overall, these findings highlight the high specificity of the model and its potential applicability in virtual screening workflows aimed at identifying selective PI3K inhibitors.

**Table 5:** List of Datasets with Non-PI3K Binders, Selected from Experimental Assays

protein	compounds	inactive (%)	active (%)
acetylcholinesterase	8127	99.8	0.2
adenosine A2a receptor	1977	98.1	1.9
aldose reductase	1163	99.7	0.3
androgen receptor	3707	97.7	2.3
angiotensin converting enzyme	876	100	0.0
caspase 3	2434	98.8	1.2
cytochrome P450 2C19 (CYP2C19)	3397	96.1	3.9
cytochrome P450 2C9 (CYP2C9)	5366	99.7	0.3
cytochrome P450 3A4 (CYP3A4)	11165	95.5	4.5
copamine D3	508	99.8	0.2
epidermal growth factor receptor (EGFR)	16711	97.9	2.1
estrogen receptor alpha	4251	99.6	0.4
fibroblast growth factor receptor 1 (FGFR1)	3222	94.8	5.2
gamma-aminobutyric acid receptor alpha-1	40	100	0.0
HIV-1 reverse transcriptase	10104	99.0	1.0
insulin receptor	1364	98.5	1.5
RAS	688	89.7	10.3
renin	3083	99.0	1.0
serine/threonine protein kinase B RAF	4930	96.0	4.0
xanthine oxidase	602	99.0	1.0

## 4 Conclusions

In this work, we present the PI3K-Seeker server, a powerful and freely accessible computational tool designed to speed up drug discovery by narrowing down compounds with the ability to bind to the ATP-binding pocket of PI3K class I enzymes. The server integrates the use of two machine learning models based on the XGB algorithm to differentiate between PI3K binders and nonbinders. The user can easily interrogate the server by solely supplying a list of SMILES strings. Compared to existing approaches, the PI3K-Seeker server offers superior performance, delivering high-accuracy predictions in under seconds. This study highlights the predictive power of machine learning algorithms in virtual screening protocols, contributing to speeding up the discovery of PI3K inhibitors.

## Supplementary Material









## Data Availability

The PI3K-Seeker server is freely available at http://www.ufrgs.br/labec/pi3k-seeker/.

## Notes

Machine learning algorithms and hyperparameters (Table S1); metrics for all datasets, fingerprints, and machine learning algorithms in the test set (Table S2); and physicochemical properties (Figure S1) (PDF)
Dataset1.csv (2.74 MB) (ZIP)
Dataset2.csv (714 KB) (ZIP)
Dataset3.csv (1.42 MB) (ZIP)
