# Supplementary material for: PI3K-Seeker: A Machine Learning-Powered Web Tool to Discover PI3K Inhibitors
Source: ACS Omega. 2025 Nov 18;10(47):57255–66. doi: 10.1021/acsomega.5c07315 (PMC12676366; doi:10.1021/acsomega.5c07315)
Supplement: Supplementary file 1 [file ao5c07315_si_001.pdf]

Supplementary material

Title: "PI3K-Seeker: A machine learning-powered web tool to discover PI3K inhibitors"

Francisca Joseli Freitas de Sousa[§], *Dinler Amaral Antunes*[‡] *and Geancarlo Zanatta*[†§*]
[§]Postgraduate Programme in Biochemistry, Department of Biochemistry at Federal University of Ceará, Fortaleza 60440-554, CE, Brazil.
[‡]Department of Biology and Biochemistry, University of Houston, Texas, USA.
[†]Department of Biophysics, Federal University of Rio Grande do Sul, Porto Alegre, Brazil.
*Corresponding author: geancarlo.zanatta@gmail.com

Table S1 - Machine learning algorithms and hyperparameters

Model	Type	Hyperparameters
SVM	Kernel function	Kernel = 'rbf'
		Gamma = 0.05
		C = 3
		Probability = TRUE
		Cross_validation = 10
GAT	Deep Learning	Hidden_dim = 32
		Heads = 4
		Dropout = 0.2
		Learning rate = 0.0005
		Epochs = 50
		Batch_size = 64
RF	Ensemble learning	N_estimators = 1500
		Class_weight = ‘balanced'
		Cross_validation = 10
XGB	Ensemble learning	N_estimators = 1500
		Eta = 0.05
		Cross_validation = 10

Legend: SVM (Support Vector Machine); RF (Random Forest); XGB (eXtreme Gradient Boosting); GAT (Graph Attention Network).

Table S2. Metrics for all datasets, fingerprints, and machine learning algorithms in the test set.

Algorithm	Dataset	Fingerprint	ACC	MCC	F1-score	Precision	Recall	AUC
SVM	Dataset1	Estate	0.883	0.649	0.716	0.797	0.651	0.920
	Dataset2		0.896	0.312	0.944	0.898	**0.996**	0.848
	Dataset3		**0.935**	**0.865**	**0.919**	**0.898**	0.941	**0.972**
	Dataset1	MACCS	0.963	0.893	0.918	0.919	0.916	0.987
	Dataset2		0.939	0.669	**0.966**	**0.945**	**0.989**	0.909
	Dataset3		**0.968**	**0.933**	0.959	0.942	0.977	**0.991**
	Dataset1	PubChem	0.972	0.919	0.938	0.941	0.935	0.990
	Dataset2		0.936	0.656	0.965	0.946	**0.984**	0.945
	Dataset3		**0.974**	**0.946**	**0.967**	**0.963**	0.972	**0.992**
RF	Dataset1	Estate	0.907	0.749	0.807	0.765	0.854	0.955
	Dataset2		0.894	0.489	**0.940**	**0.942**	0.938	0.989
	Dataset3		**0.945**	**0.887**	0.932	0.916	**0.948**	**0.992**
	Dataset1	MACCS	0.962	0.890	0.914	0.927	0.902	0.891
	Dataset2		0.929	0.614	**0.960**	0.943	**0.978**	0.928
	Dataset3		**0.965**	**0.926**	0.955	**0.946**	0.965	**0.951**
	Dataset1	PubChem	0.968	0.907	0.928	0.941	0.915	0.981
	Dataset2		0.935	0.649	**0.964**	0.945	**0.984**	**0.993**
	Dataset3		**0.968**	**0.933**	0.960	**0.951**	0.968	**0.993**
XGB	Dataset1	Estate	0.911	0.740	0.796	0.830	0.764	0.959
	Dataset2		0.922	0.573	**0.957**	**0.937**	**0.978**	0.917
	Dataset3		**0.947**	**0.890**	0.934	0.917	0.951	**0.984**
	Dataset1	MACCS	**0.965**	0.899	0.922	0.926	0.917	0.990
	Dataset2		0.929	0.617	**0.960**	**0.944**	**0.977**	0.929
	Dataset3		0.964	**0.925**	0.955	0.943	0.966	**0.993**
	Dataset1	PubChem	**0.971**	0.919	0.937	0.939	0.936	**0.993**
	Dataset2		0.935	**0.658**	0.964	0.951	0.977	0.951
	Dataset3		**0.971**	**0.937**	**0.962**	**0.952**	**0.972**	**0.993**
GAT	Dataset1	Estate	0.756	0.428	0.572	0.476	0.716	0.820
	Dataset2		0.709	0.223	0.815	**0.931**	0.724	0.754
	Dataset3		**0.901**	**0.801**	**0.881**	0.833	**0.935**	**0.957**
	Dataset1	MACCS	0.842	0.627	0.713	0.606	0.866	0.922
	Dataset2		0.746	0.341	0.839	**0.956**	0.747	0.824
	Dataset3		**0.936**	**0.867**	**0.920**	0.894	**0.949**	**0.975**
	Dataset1	PubChem	0.842	0.630	0.715	0.607	0.871	0.922
	Dataset2		0.835	0.471	0.900	**0.965**	0.844	0.878
	Dataset3		**0.950**	**0.896**	**0.937**	0.921	**0.955**	**0.985**

Legend: SVM (Support Vector Machine); RF (Random Forest); XGB (eXtreme Gradient Boosting); GAT (Graph Attention Network); ACC (Accuracy); MCC (Matthews Correlation Coefficient); AUC (Area under the curve).

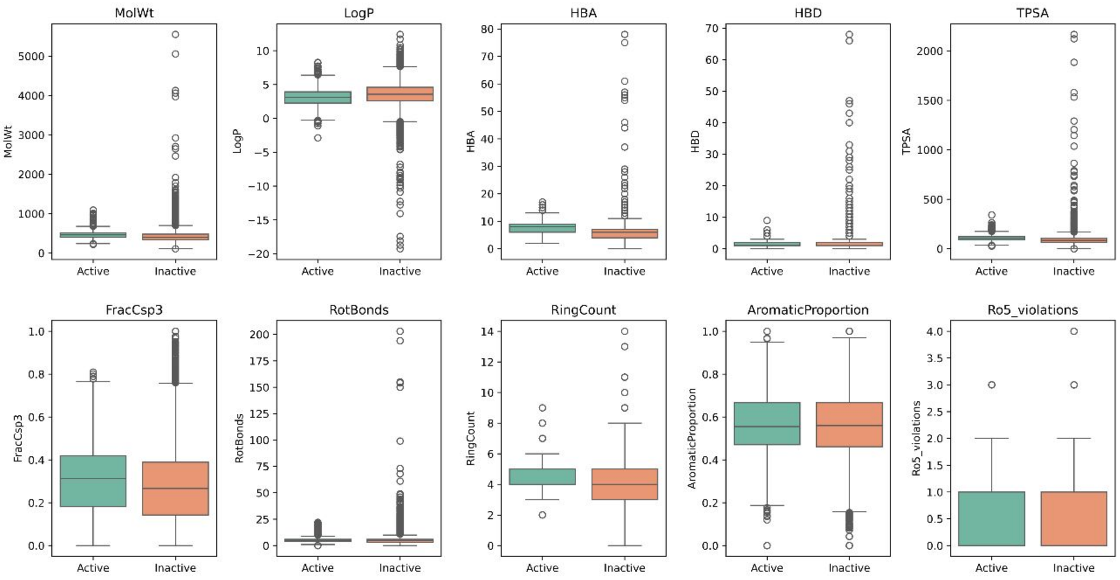

Figure S1 – Physicochemical properties.
